# DNA Aptamers against Taiwan Banded Krait α-Bungarotoxin Recognize Taiwan Cobra Cardiotoxins

**DOI:** 10.3390/toxins8030066

**Published:** 2016-03-05

**Authors:** Ying-Jung Chen, Chia-Yu Tsai, Wan-Ping Hu, Long-Sen Chang

**Affiliations:** 1 Institute of Biomedical Sciences, National Sun Yat-Sen University, Kaohsiung 804, Taiwan; yjchen@mail.nsysu.edu.tw (Y.-J.C.); m012050015@student.nsysu.edu.tw (C.-Y.T.); 2 Department of Biotechnology, Kaohsiung Medical University, Kaohsiung 807, Taiwan; wphu@kmu.edu.tw

**Keywords:** aptamer, α-bungarotoxin, cardiotoxins, membrane-damaging activity, cytotoxicity

## Abstract

*Bungarus multicinctus* α-bungarotoxin (α-Bgt) and *Naja atra* cardiotoxins (CTXs) share a common structural scaffold, and their tertiary structures adopt three-fingered loop motifs. Four DNA aptamers against α-Bgt have been reported previously. Given that the binding of aptamers with targeted proteins depends on structural complementarity, in this study, we investigated whether DNA aptamers against α-Bgt could also recognize CTXs. It was found that *N.* *atra* cardiotoxin 3 (CTX3) reduced the electrophoretic mobility of aptamers against α-Bgt. Analysis of the changes in the fluorescence intensity of carboxyfluorescein-labeled aptamers upon binding toxin molecules revealed that CTX3 and α-Bgt could bind the tested aptamers. Moreover, the aptamers inhibited the membrane-damaging activity and cytotoxicity of CTX3. In addition to CTX3, other *N. atra* CTX isotoxins also bound to the aptamer against α-Bgt. Taken together, our data indicate that aptamers against α-Bgt show cross-reactivity with CTXs. The findings that aptamers against α-Bgt also suppress the biological activities of CTX3 highlight the potential utility of aptamers in regard to the broad inhibition of snake venom three-fingered proteins.

## 1. Introduction

Aptamers are synthetic oligonucleotides, such as RNA and single-stranded DNA, that can bind to their targets with high affinity and specificity due to their specific secondary or tertiary structures [1,2]. A number of studies have suggested the utility of aptamers as diagnostic tools, therapeutic, drug delivery, and biomarker discovery agents, bioimaging tools, and biosensor probes [2]. Although aptamers differ from antibodies, they mimic the properties of antibodies in a wide range of biological applications. Moreover, the heat-stability, low immunogenicity, and target variants of aptamers can overcome the disadvantages of antibodies [1].

Snake venom contains a number of pharmacologically-active proteins. Conventional antivenoms are prepared from the sera of horses or sheep after they are hyperimmunized with the relevant snake venom [3]. Although anti-snake venom antibodies are wildly used and effective for treating snake bites, the immunization of horses or other animals with crude venoms for antivenom production is difficult due to the highly lethal or toxic character of the venom proteins. Moreover, products composed of antibodies from immunized animals may cause adverse reactions in humans due to activation of the immune system [4]. Progressive injuries including deteriorating local injuries (e.g., swelling, ecchymosis), coagulation abnormalities, or systemic effects (e.g., hypotension, altered mental status) have been reported upon administration of some antivenoms [5]. Thus, aptamers might overcome the disadvantages and limitations of conventional antivenoms. In recent studies, aptamers against *Bungarus multicinctus* α-bungarotoxin (α-Bgt), and β-bungarotoxin (β-Bgt) were produced [6,7]. Aptamers against β-Bgt can discriminate β-Bgt from other tested snake venom proteins [7]. Four aptamers against α-Bgt were identified from bound carboxyfluorescein-labeled oligonucleotides on an α-Bgt-coated glass coverslip, but only one aptamer showed the binding capabilities with α-Bgt when the aptamer-α-Bgt interaction was analyzed using surface plasma resonance (SPR) [6]. β-Bgt is a long α-neurotoxin and its three-dimensional structure adopts a three-fingered loop-folding topology dominated with a,β-sheet [8]. The tertiary structures of snake venom cardiotoxins (CTXs), short α-neurotoxins, and neurotoxin homologues also adopt three-loop motifs, but differ in the extent of their secondary structure and positioning of the invariant side chains [9,10,11,12]. Sequence alignments of long α-neurotoxins, CTXs, short α-neurotoxins, and neurotoxin homologues revealed that these proteins share sequence similarities and their cysteine residues are located at consensus positions [10,13]. Moreover, analyses of the genetic structures indicate that long α-neurotoxin, CTXs, short α-neurotoxins and neurotoxin homologues share a common evolutionary origin [13,14]. Previous studies showed that aptamer-binding by proteins was largely determined by how well the molecules fit into the cavities of the target proteins [15,16]. Moreover, species cross-reactivity of aptamers with orthologous proteins was also reported [17,18]. Taken together, one may wonder whether aptamers against α-Bgt can bind three-fingered snake venom proteins owing to complementary molecular surface. To address that question, the interactions between aptamers against α-Bgt and *Naja atra* (Taiwan cobra) cardiotoxin 3 (CTX3) were analyzed in the present study.

## 2. Results and Discussion

**Figure 1:**
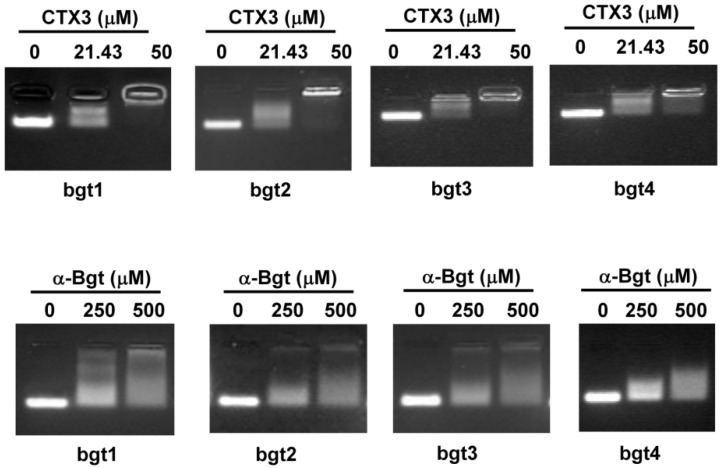
Electrophoretic mobility shift assay of the binding of bgt1, bgt2, bgt3, and bgt4 aptamers to CTX3 and α-Bgt: 5 μM aptamers against α-Bgt was incubated with indicated concentrations of CTX3 and α-Bgt for 20 min, and then the aptamer-toxin mixtures were separated on 2% agarose gel.

Four DNA aptamers against α-Bgt were reported previously [6]. The four aptamers are designated as bgt1, bgt2, bgt3, and bgt4 in the present study (Table 1). To investigate the binding of aptamers with CTX3 and α-Bgt, an electrophoretic mobility shift assay was conducted. As shown in Figure 1, toxin-aptamer complexes were formed when 21.43 μM CTX3 was incubated with 5 μM of the aptamers, indicating that CTX3 reduced the electrophoretic mobility of the aptamers. When incubated with 50 μM CTX3, the aptamer DNA mostly stuck in the sample well. Toxin-aptamer complexes also formed when 250 μM α-Bgt was incubated with 5 μM of the aptamers. When the concentration of α-Bgt was increased to 500 μM, the migration of DNA aptamers into agarose gels could not be completely inhibited. These observations suggested that CTX3 and α-Bgt could bind the tested aptamers. Moreover, compared to α-Bgt, CTX3 more readily reduced the electrophoretic mobility of the aptamers.

**Figure 2:**
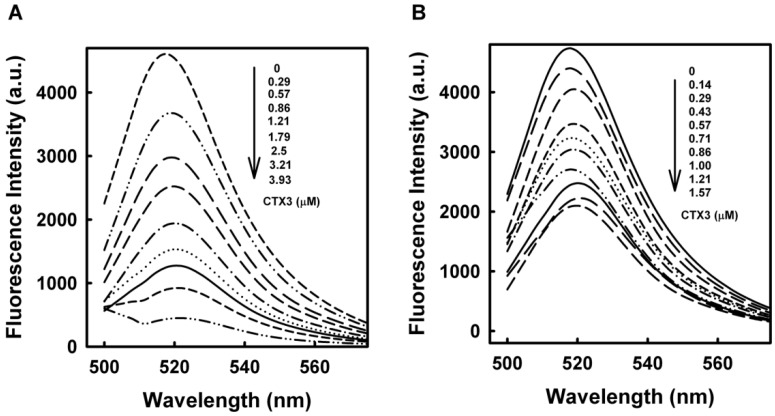
Fluorescence spectra of FAM-bgt1-DABCYL and FAM-bgt1 in the presence of various CTX3 concentrations as indicated: (**A**) FAM-bgt1-DABCYL and (**B**) FAM-bgt1 in 10 mM Tris-HCl (pH 7.5) containing 1 mM EDTA and 100 mM NaCl were titrated with CTX3.

To determine the binding affinity of CTX3 and α-Bgt with bgt1-4, the aptamers were functionalized with a FAM reporter at the 5′-end and a DABCYL quencher at the 3′-end. Previous studies suggested that binding to the target molecule induced a conformational change in aptamers [19,20]. If binding CTX3 or α-Bgt could induce a conformational change, the protein-bound aptamer conformation may bring FAM and DABCYL into close proximity, resulting in quenched fluorescence of FAM. On the other hand, previous studies also revealed that the proximity of guanine to FAM could quench the fluorescence [21]. The guanine content in bgt1–4 ranged from 31% to 38%. Thus, aptamers with a FAM reporter at the 5′-end were also synthesized. Given that bgt1 was the only aptamer against α-Bgt that showed binding capabilities with α-Bgt using SPR analyses [6], the effect of CTX3 on the fluorescence intensity of FAM-bgt1 and FAM-bgt1-DABCYL was analyzed. As shown in Figure 2, the binding of CTX3 with FAM-bgt1-DABCYL caused a reduction in the fluorescence intensity at 520 nm. Reduction in FAM fluorescence intensity was maximized when the CTX3 concentration was >3.93 μM. On the other hand, binding of CTX3 with FAM-bgt1 also caused a reduction in FAM fluorescence intensity, suggesting that the FAM group might move towards guanine in toxin-bound FAM-bgt1. In view of the findings that CTX3 caused a greater reduction in the fluorescence intensity of FAM-bgt1-DABCYL than that of FAM-bgt1, the CTX-induced reduction in fluorescence intensity of FAM-bgt1-DABCYL was attributed to the quenching of FAM fluorescence by the DABCYL group and guanine.

**Figure 3:**
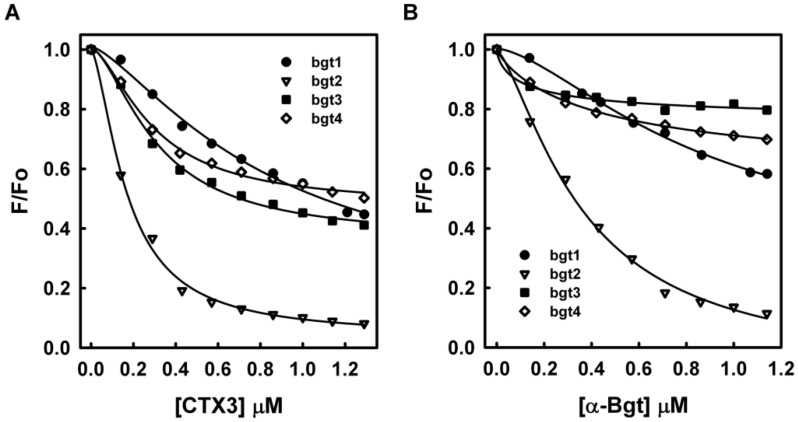
Effect of CTX3 and α-Bgt on fluorescence intensity of FAM-labeled bgt1, bgt2, bgt3, and bgt4 aptamers: the fluorescence intensity of FAM-labeled bgt1, bgt2, bgt3 and bgt4 was determined at 520 nm in the presence of various concentrations of (**A**) CTX3 or (**B**) α-Bgt. Fo and F represent the fluorescence intensity of FAM-labeled bgt1, bgt2, bgt3, and bgt4 in the absence and presence of toxins.

The dissociation constants of CTX3 with FAM-bgt1 and FAM-bgt1-DABCYL were calculated using the titration data derived from the change in FAM fluorescence intensity induced by toxin molecules. The *K*_d_ values of CTX3 with FAM-bgt1 and FAM-bgt1-DABCYL were 2.25 μM and 2.35 μM. Since CTX3 showed similar binding affinities towards FAM-bgt1 and FAM-bgt1-DABCYL, the binding capabilities of CTX3 and α-Bgt with DNA aptamers were analyzed using FAM-labeled aptamers in the following experiments. To prove the reduction in fluorescence only occurred upon binding of CTX3 or α-Bgt to the oligonucleotides, a control experiment was conducted by titrating a FAM solution with CTX3 or α-Bgt. CTX3 or α-Bgt did not significantly affect the fluorescence intensity of the FAM solution. Thus, any change in the fluorescence intensity of the FAM-labeled aptamers was caused by the binding of the toxins to the aptamers. As shown in Figure 3, titration of FAM-labeled aptamers with CTX3 or α-Bgt resulted in a reduction in FAM fluorescence intensity. The *K*_d_ values of CTX3 for bgt2, bgt3, and bgt4 calculated from the changes in fluorescence intensity were 0.26 μM, 1.26 μM, and 1.17 μM, respectively. The *K*_d_ values of α-Bgt for bgt1, bgt2, bgt3, and bgt4 were 2.21 μM, 0.46 μM, 0.14 μM, and 0.28 μM, respectively. Unlike the study conducted by Lauridsen *et al.* [6], which showed that α-Bgt only bound with the bgt1 aptamer, our data revealed that all four aptamers could bind α-Bgt. Notably, Lauridsen *et al.* [6] analyzed the binding between α-Bgt and DNA aptamers using biotinylated aptamers that were immobilized on a streptavidin-coated chip. Presumably, steric hindrance caused by the biotin-streptavidin interaction blocked the binding of bgt2, bgt3, and bgt4 by α-Bgt. SPR analyses also revealed that the dissociation constant of α-Bgt for bgt1 was 7.5 μM [6], which was similar to that measured using FAM-bgt1 in the present study. These results confirmed the measurements of the aptamer-toxin interaction based on changes in the fluorescence intensity of FAM-labeled bgt1–4.

**Figure 4:**
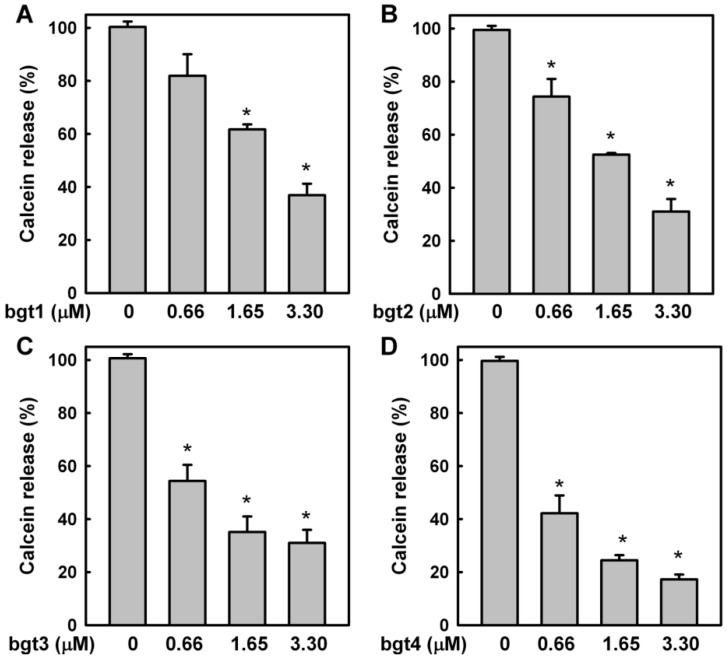
Effect of bgt1 (**A**), bgt2 (**B**), bgt3 (**C**), and bgt4 (**D**) aptamers on membrane-damaging activity of CTX3: CTX3 (0.23 μM) was incubated with 0.66, 1.65, and 3.30 μM aptamers against α-Bgt for 20 min, and then membrane-damaging activity of CTX3-aptamer mixtures were measured. Data represents mean ± SD of three independent experiments with triplicated measurements (* *p* *<* 0.05, *vs.* CTX3 without incubation with aptamers). The experiments were performed in 10 mM Tris-HCl (pH 7.5) containing 1 mM EDTA and 100 mM NaCl. The signal was expressed as the percentage of total calcein release after addition of 0.2% Triton X-100.

**Figure 5:**
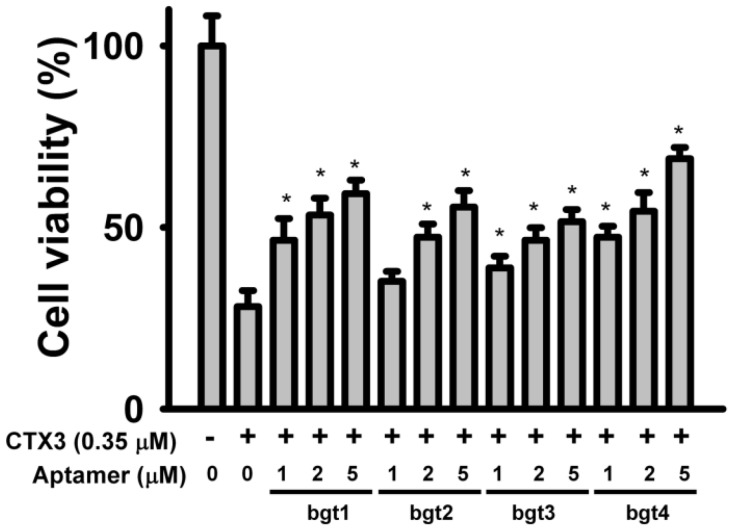
Effect of bgt1, bgt2, bgt3, and bgt4 aptamers on the cytotoxcity of CTX3: CTX3 (0.35 μM) was incubated with 1, 2, and 5 μM aptamers against α-Bgt for 20 min, and then the cytotoxicity of CTX3-aptamer mixtures were measured according to the procedure described in Materials and Methods section. Data represent mean ± SD of three independent experiments with triplicated measurements (* *p* *<* 0.05, *vs.* CTX3 without incubation with aptamers).

To analyze whether aptamer binding inhibited the biological activities of CTX3, the effects of the aptamers on the membrane-damaging activity and cytotoxicity of CTX3 were investigated. The membrane-damaging activity of CTX3 was measured by the release of calcein from lipid vesicles. As shown in Figure 4, bgt1, bgt2, bgt3, and bgt4 inhibited the membrane-damaging activity of CTX3 in a concentration-dependent manner. Compared to bgt1, bgt2, and bgt3, bgt4 exhibited superior inhibition of the membrane-damaging activity of CTX3. On the other hand, bgt1, bgt2, bgt3, and bgt4 also inhibited the cytotoxicity of CTX3 on K562 cells in a concentration-dependent manner (Figure 5). Compared to that by 5 μM of bgt1, bgt2, and bgt3, the cytotoxicity of 0.35 μM CTX3 was inhibited by 5 μM bgt4 to a greater extent.

**Figure 6:**
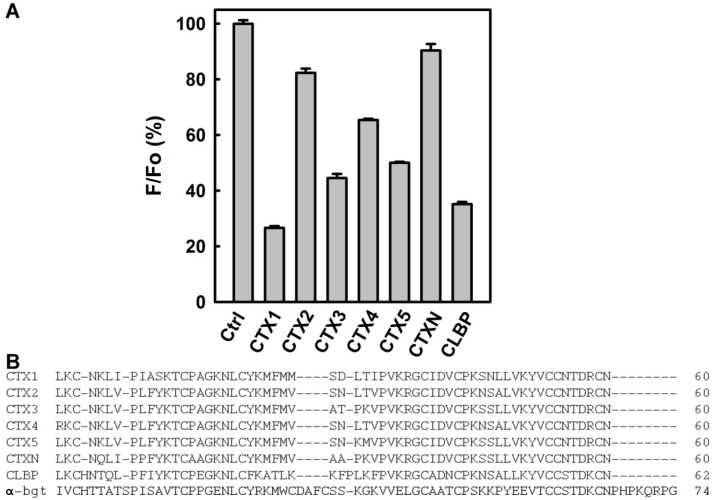
The fluorescence intensity of FAM-bgt1 in the presence of CTX isotoxins: (**A**) Fo and F represent the fluorescence intensity of FAM-bgt1 in the absence and presence of 1.5 μM CTXs. The fluorescence intensity was measured at 520 nm; and (**B**) amino acid sequence alignments of CTX1, CTX2, CTX3, CTX4, CTX5, CTXN, CLBP, and α-Bgt.

Previous studies revealed that *N. atra* CTX1, CTX2, CTX4, CTX5, CTXN, and CLBP were structurally homologous to CTX3 [11,22,23]. Thus, the interactions between bgt1 and CTX1, CTX2, CTX4, CTX5, CTXN, and CLBP were also analyzed. As shown in Figure 6A, CTX isotoxins reduced the fluorescence intensity of bgt1 to different extents, revealing that CTX isotoxin binding affected the conformation of bgt1 in different ways. The dissociation constants (*K*_d_) of CTX1, CTX2, CTX4, CTX5, CTXN, and CLBP towards bgt1 were 2.51 μM, 6.29 μM, 8.13 μM, 17.17 μM, 8.85 μM, and 7.19 μM, respectively.

Our data show that the aptamers against α-Bgt exhibit cross-reactivity with *N. atra* CTXs, suggesting that aptamers against α-Bgt prefer to interact with structural scaffolds with three-fingered loop structures. Although the binding affinity of α-Bgt with the tested aptamers was similar to or even greater than that of CTX3 (Table 1), the ability of α-Bgt to inhibit the electrophoretic migration of aptamers was weaker than that of CTX3 (Figure 1). The pIs (isoelectric point) of CTX3 and α-Bgt are >10 and 8.4, respectively [24,25]. Considering that the pH of agarose electrophoretic TAE (Tris-Acetate-EDTA) buffer is approximately 8.0, the distinct effects of CTX3 and α-Bgt on the electrophoretic mobility of aptamers could be attributed to the differences in their net positively charges, which influences their interactions with the negatively charged phosphate groups of DNA aptamers. CTX3 showed a higher binding affinity towards bgt2 than bgt1, bgt3, and bgt4, but the inhibitory effect of bgt4 on the membrane-damaging activity and cytotoxicity of CTX3 was greater than that of bgt1, bgt2, and bgt3. These results imply that the interaction between bgt4 and CTX3 is distinct from that of bgt1, bgt2, and bgt3. A number of studies have been conducted to elucidate the structure-function relationship of CTXs by chemical modifications and site-directed mutagenesis [26,27,28,29,30]. These studies suggested that no single-loop region was exclusively responsible for the biological activities of CTXs. Thus, it is conceivable that bgt1, bgt2, bgt3, and bgt4 all effectively suppress the biological activities of CTX3 even though the DNA aptamers may adopt different binding modes with CTX3. Sequence alignments reveal that α-Bgt and CTXs share low sequence identity (Figure 6B). Likewise, the amino acid sequence of CLBP does not show a high degree of homology with those of CTXs. Nevertheless, CLBP exhibits a higher binding affinity for bgt1 than CTX4, CTX5, and CTXN, and the binding affinity of α-Bgt wth bgt1 is higher than that of CTXs. These results likely suggest that the binding of bgt1 with α-Bgt, CTXs, and CLBP largely depends on complementary molecular surface rather than specific amino acid residues.

In summary, our data suggest that aptamers against α-Bgt may broadly inhibit the biological activities of snake venom three-fingered proteins. However, the binding of CTX3 and α-Bgt towards aptamers exhibited μM-level dissociation constants. Compared to other therapeutic aptamers with nM binding affinities towards their targets [1], improvement in the aptamer binding capabilities represents a crucial step before the utility of aptamers as inhibitors of snake venom three-fingered protein toxicity reaches its full potential. Previous studies showed that high-affinity aptamers with thrombin have been successfully screened using computational modeling or bivalent aptamer [31,32]. This may be a feasible strategy in improving the aptamer binding capabilities against snake venom three-fingered proteins.

## 3. Materials and Methods

Crude venoms of *Naja naja* (Taiwan cobra) from different geographic area in Taiwan are pooled together for purification of CTX isotoxins. CTX isotoxins including CTX1, CTX2, CTX3, CTX4, CTX5, CTXN, and cardiotoxin-like protein (CLBP) were isolated from the venom of *N. atra* according to the procedure described in Lin *et al.* [33]. Purification of α-Bgt from *Bungarus multicinctus* (Taiwan banded krait) venom was carried out according to the procedure described in Chang *et al.* [14]. Calcein, carboxyfluorescein (FAM), egg yolk phosphatidylcholine (EYPC), dimyristoyl phosphatidic acid (DMPA), and MTT were purchased from Sigma-Aldrich Inc., St. Louis, MO, USA, and 5′-carboxyfluorescein (FAM)-labeled single-stranded DNA or 5′-FAM/3′-4-([4-(dimethylamino)- phenyl]azo)-benzoic acid (DABCYL)-labeled single-stranded DNA samples were synthesized from Neogene Biomedicals Corporation (Taipei, Taiwan). Unless otherwise specified, all other reagents were analytical grade.

### 3.1. Electrophoretic Mobility Shift Assay

Aptamers (5 μM) against α-Bgt dissolved in 10 mM Tris-HCl (pH 7.5) containing 1 mM EDTA and 100 mM NaCl were incubated with CTX3 and α-Bgt for 20 min. The protein-DNA mixtures were then analyzed by 2% agarose gels.

### 3.2. Fluorescence Measurement of the Binding of CTXs and α-Bgt with Apatmers

All samples were prepared in solution containing 10 mM Tris-HCl (pH 7.5), 1 mM EDTA and 100 mM NaCl. The FAM-apatmer-DABCYL (4 nM) and FAM-aptamer (4 nM) were titrated with small aliquots of CTXs or α-Bgt. Total dilution never exceeded 10% and the relative fluorescence values were uniformly corrected for dilution. Titration of CTXs and α-Bgt was stopped when fluorescence intensity of FAM-aptamer and FAM-aptamer-DABCYL was no more decreased. The fluorescence spectra were measured on a Hitachi F-4500 Fluorescence spectrophotometer (Hitachi High Technologies Co., Tokyo, Japan) with excitation and emission wavelengths at 480 and 520 nm, respectively. A plot of the 1/(Fo-F) *versus* 1/[Toxin] gives lines with a slope corresponding to the dissociation constant of aptamer-toxin complexes. Fo and F are fluorescence intensities in the absence or presence of toxins.

### 3.3. Release of Entrapped Fluorescent Marker from Liposomes

EYPC/DMPA at a molar ratio of 9:1 was dissolved in chloroform/methanol (*v*/*v*, 2:1) and dried by evaporation. Buffer (10 mM Tris-HCl, 1 mM EDTA, and 100 mM NaCl, pH 7.5) containing 50 mM calcein was added to the film of lipids, and after hydration the suspension was frozen and thawed several times. The multilamellar vesicles were extruded 10 times, above the transition temperature, through a 100-nm polycarbonate filter and applied to a Sepharose 6B column (2 × 15 cm) to separate the liposome from the free calcein. Leakage was induced by adding aliquots of CTX3 to a vesicle suspension directly in the cuvette used for fluorescence determination at 30 °C. The kinetics of membrane damage were monitored by the increase in fluorescence with emission at 520 nm and excitation at 490 nm, and the signal was expressed as percentage of total calcein release after the addition of 0.2% Triton X-100.

### 3.4. Cell Viability Assay

Human leukemia K562 cells were obtained from ATCC (Rockville, MD, USA). K562 cells were cultured in RPMI 1640 medium supplemented with 10% fetal calf serum (Gibco BRL, Grand island, NY, USA), 2 mM l-glutamine, penicillin (100 U/mL)/streptomycin (100 μg/mL), and 1% sodium pyruvate in an incubator humidified with 95% air and 5% CO_2_. Exponentially growing cells (1 × 10^5^) were plated in 96-well plates and treated with CTX3 in serum-free medium for 3 h. Then MTT solution was added to each well at a final concentration of 0.5 mg/mL and incubated for 4 h. Formazan crystals resulting from MTT reduction were dissolved by addition of 100 μL DMSO per well. The absorbance was detected at 595 nm using a plate reader.

### 3.5. Statistical Analysis

All data are presented as mean ± SD. Significant differences among the groups were determined using the unpaired Student’s *t*-test. A value of *p* < 0.05 was taken as an indication of statistical significance.

## Figures and Tables

**Table 1:** The dissociation constant of CTX3 and α-Bgt with aptamer against α-Bgt.

Aptamer ^1^	CTX3 *K*_d_ (μM)	α-Bgt *K*_d_ (μM)
bgt1	2.25	2.21
bgt2	0.26	0.46
bgt3	1.26	0.14
bgt4	1.17	0.28

^1^ The nucleotide sequences of bgt1, bgt2, bgt3, and bgt4 are 5′-GCGAGGTGTTCGAGAGTTAGGGCGACATGACCAAACGTT-3′, 5′-AGGGCACAGAGAAGAAGTCGTGGATTTGAATGGTTTTGGT-3′, 5′-ATCATGTCTTTTCGGGATGGGCAAGAAGGGAAATAATGC-3′ and 5′-AGAAACGTAGCGGTAACTGCTAGAATGCGCCGAGAGAGCG-3′, respectively.

## Abbreviations

The following abbreviations are used in this manuscript:

α-Bgt: α-Bungarotoxin
CTX: Cardiotoxin
CLBP: Cardiotoxin-like protein
DMPA: Dimyristoyl phosphatidic acid
DABCYL: 4-([4-(dimethylamino)phenyl]azo)-benzoic acid
EYPC: Egg yolk phosphatidylcholine
FAM: Carboxyfluorescein
